# Psychological distress and its influencing factors in esophageal cancer patients undergoing radiotherapy: a longitudinal study

**DOI:** 10.3389/fonc.2025.1582870

**Published:** 2025-11-07

**Authors:** Jin Li, Xiaofan Li, Jue Chen, Ying Zhang, Ying Yao, Huimin Yang

**Affiliations:** 1 Department of Radiation Oncology, The First Affiliated Hospital of Henan University of Science and Technology, Luoyang, Henan, China; 2 School of Nursing, Henan University of Science and Technology, Luoyang, Henan, China; 3 Department of Respiratory and Critical Care Medicine, The First Affiliated Hospital of Henan University of Science and Technology, Luoyang, Henan, China; 4 Department of Scientific Research, The First Affiliated Hospital of Henan University of Science and Technology, Luoyang, Henan, China

**Keywords:** psychological distress, esophageal cancer, radiotherapy, influencing factors, prospective study

## Abstract

**Objective:**

To investigate the dynamic changes of psychological distress and its influencing factors in cancer patients across different phases of radiotherapy.

**Methods:**

Using a convenience sampling method, 226 esophageal cancer patients receiving radiotherapy at the Oncology Radiotherapy Center of a tertiary (Grade‐A) hospital in Henan Province were recruited from September 2022 to October 2023. Psychological distress was assessed using the Distress Thermometer (DT) at four time points: before the first radiotherapy session (T1), at the 15th session (T2), at the end of radiotherapy (T3), and one month after the completion of radiotherapy (T4). Generalized Estimating Equations (GEE) were utilized to analyze the factors related to psychological distress.

**Results:**

Among the 199 patients who completed the longitudinal follow-up, the mean psychological distress scores were 4.88 ± 1.63(T1); 5.09 ± 1.57(T2); 4.75 ± 1.56(T3); and 4.06 ± 1.57 (T4) respectively. GEE results indicated that age, monthly household income per capita, tumor stage, concurrent chemoradiotherapy, symptom burden, family support, and illness perception were influencing factors of psychological distress in esophageal cancer patients undergoing radiotherapy (P < 0.05).

**Conclusion:**

Esophageal cancer patients exhibit moderate psychological distress during radiotherapy, with a trend of initial increase followed by a decrease as radiotherapy continues. Based on the changing characteristics and identified influencing factors, the healthcare team should develop dynamic and individualized intervention strategies to reduce patients’ psychological distress.

## 1 Introduction

Esophageal cancer remains a major global public health challenge. According to the 2020 Global Cancer Statistics, it ranked as the sixth most commonly diagnosed cancer worldwide, with approximately 604, 000 new cases and 544, 000 deaths annually. China bears a particularly high burden, accounting for 324, 000 new cases and 301, 000 deaths-representing 53.70% and 55.35% of global incidence and mortality, respectively (1). These epidemiological data highlight the urgent need for effective and comprehensive management strategies for esophageal cancer, particularly in high-incidence regions.

The management of esophageal cancer typically follows a multimodal approach that integrates surgery, chemotherapy, and radiotherapy. For patients with locally advanced or inoperable disease, radiotherapy constitutes a cornerstone of both curative and palliative treatment (2). Advances in radiotherapy techniques, such as intensity-modulated radiotherapy (IMRT), have enhanced targeting precision and reduced damage to adjacent healthy tissues. Nevertheless, despite these technical improvements, radiotherapy remains associated with considerable treatment-related toxicities, including radiation esophagitis, dermatitis, fatigue, pneumonitis, and nutrition-related weight loss (2, 3). These adverse effects not only impair physical health and quality of life but also significantly contribute to psychological distress (4).

Psychological distress-a multidimensional unpleasant experience stemming from various sources-can compromise patients’ ability to cope with cancer and adhere to treatment, potentially leading to unfavorable outcomes (5). It has also been closely linked to postoperative quality of life and coping mechanisms (6–8). The rigorous process of radiotherapy, with its accumulating side effects and disruption of daily activities, can exacerbate this distress, often resulting in clinically significant anxiety, depression, and diminished psychological well-being (9–13). If left unaddressed, psychological distress is associated with poorer potentially worse survival outcomes.

Despite growing recognition of the biopsychosocial model in oncology, the longitudinal progression of psychological distress among esophageal cancer patients undergoing radiotherapy remains insufficiently elucidated. Current evidence on cancer-related distress suffers from limitations that constrain its applicability to this specific population. A predominant reliance on cross-sectional methodologies yields static assessments that cannot capture the temporal dynamics of distress throughout the treatment continuum. Moreover, the frequent aggregation of data from heterogeneous cancer cohorts masks the distinctive psychosocial profile of esophageal cancer patients. The condition’s characteristic symptomatology-notably dysphagia, which profoundly impacts nutritional intake and social interaction-combined with the particular toxicities associated with thoracic irradiation, generates a psychosocial burden qualitatively and quantitatively distinct from other malignancies. Potential influencing factors span multiple domains, encompassing sociodemographic attributes (e.g., age, educational attainment, socioeconomic status), clinical parameters (e.g., tumor stage, treatment protocol, toxicity profiles), and psychosocial assets (e.g., social support networks, coping mechanisms) (14). A longitudinal study that concurrently evaluates these variables is essential to identify modifiable risk and protective factors.

Consequently, this study aims to characterize the longitudinal course of psychological distress and identify influencing factors in esophageal cancer patients undergoing radiotherapy. The resulting insights are anticipated to underpin the development of personalized supportive care strategies, enabling timely integration of psychosocial support into the radiotherapy pathway.

## 2 Subjects and methods

### 2.1 Participants

Using a convenience sampling method, esophageal cancer patients receiving radiotherapy at the Oncology Radiotherapy Center of a tertiary (Grade‐A) hospital in Henan Province were recruited from September 2022 to October 2023.

Inclusion criteria (1): Age ≥ 18 years (2); Receiving intensity‐modulated radiotherapy (IMRT) with a dose of 1.8–2.0 Gy per session, with at least 20 sessions (3); No cognitive impairment and able to cooperate with the study. Exclusion criteria (1): History of radiotherapy (2); Severe dysfunction of major organs (3); Severe visual or hearing impairment (4); History of psychiatric disorders. Dropout criteria (1): Voluntary withdrawal (2); Discontinuation of radiotherapy or death during the study.

Sample size was estimated using G*Power 3.1 software. A small effect size f = 0.14 was selected based on conservative assumptions due to the lack of prior similar studies in this specific patient population. With α = 0.05, power (1 − β) = 0.90, an average correlation coefficient ρ = 0.5, and four repeated measurements per patient, the required sample size was calculated to be 89. Allowing for up to a 20% attrition rate, the minimum required sample size was determined to be 107. This study was approved by the hospital’s Ethics Committee (Approval No.: 20230007), and all participants were provided informed consent and voluntarily participated.

### 2.2 Instruments

#### 2.2.1 General information questionnaire

Developed by the research team after literature review and expert consultation, this questionnaire includes items on: age, gender, education level, marital status, monthly household income per capita, type of medical insurance, residence, history of chemotherapy, history of surgery, tumor location, disease duration, tumor stage, concurrent chemoradiotherapy, and radiotherapy dose.

#### 2.2.2 Distress thermometer

The DT is a single-item self-report instrument designed to assess the level of psychological distress experienced during the past week, with scores ranging from 0 to 10 (5). Higher scores indicate greater levels of distress. In 2010, Zhang Yening et al. (15) validated the DT in the Chinese context. In the present study, the DT demonstrated good internal consistency, with a Cronbach’s α coefficient of 0.812.

#### 2.2.3 Chinese version of the anderson symptom inventory gastrointestinal cancer module

The MDASI-GI-C is a self-report instrument developed by Wang et al. (16) in 2010 to assess symptom burden in gastrointestinal cancer patients across various treatment modalities. The scale consists of two primary components: the Symptom Severity subscale and the Symptom Interference subscale. The Symptom Severity subscale evaluates the intensity of 13 core symptoms and 5 gastrointestinal-specific symptoms (total 18 items) over the past 24 hours. The Symptom Interference subscale assesses the degree to which these symptoms interfere with six domains of daily functioning: general activity, work, mood, walking, relationships, and enjoyment of life (6 items).

During the pilot phase of the current study, the research team observed that scores on the Symptom Interference subscale were susceptible to influence from non-symptom-related factors. Consequently, only the Symptom Severity subscale (18 items) was employed in the main study. Each item is rated on a numerical scale from 0 (“no symptom”) to 10 (“the worst imaginable severity”). The total score for this subscale is calculated as the sum of all 18 item scores, resulting in a possible range of 0 to 180, with higher scores indicating greater symptom burden. In this study, the Symptom Severity subscale demonstrated good internal consistency, with a Cronbach’s α coefficient of 0.842.

#### 2.2.4 Brief illness perception questionnaire

The BIPQ, developed by Broadbent et al. (17) in 2006, is a self-administered instrument designed to evaluate individuals’ cognitive and emotional representations of their illness. The questionnaire comprises nine items, with the first eight scored on a 0–10 scale (items 3, 4, and 7 are reverse-scored). The ninth item is an open-ended question assessing perceived illness causes. A total score is derived from the sum of the first eight items, with higher scores indicating a more negative perception of the illness. The Chinese version was translated and validated by Na Zhang et al. (18) in 2017. In the present study, the BIPQ demonstrated good internal consistency, with a Cronbach’s α coefficient of 0.792.

#### 2.2.5 Family APGAR Index

The Family APGAR Index, originally developed by Smilkstein (19), is a brief self-report instrument designed to assess an individual’s perception of family functioning. The scale consists of five items, each rated on a 3-point scale: “Always” (2 points), “Sometimes” (1 point), and “Hardly Ever” (0 points). Total scores range from 0 to 10, with higher scores reflecting better family functioning. In the present study, the internal consistency of the scale was excellent, with a Cronbach’s α of 0.865.

### 2.3 Data collection methods

Upon receiving formal approval from the hospital and relevant departments, data collection was conducted at the Oncology Radiotherapy Center by two trained researchers. Prior to the initial radiotherapy session (T1), eligible patients were provided with a comprehensive explanation of the study objectives, significance, and procedures. Written informed consent was obtained from all participants. Patients then independently completed a set of baseline questionnaires, including the General Information Questionnaire, Distress Thermometer (DT), Symptom Severity subscale of the MDASI-GI-C, Brief Illness Perception Questionnaire (BIPQ), and the Family APGAR Index. In cases where patients were unable to complete the forms independently, the researchers administered the questionnaires through structured interviews and recorded the responses accordingly. All completed questionnaires were reviewed immediately to ensure data integrity and completeness.

Follow-up assessments were performed at the 15th radiotherapy session (T2), upon completion of radiotherapy (T3), and one month post-radiotherapy (T4). At these time points, the DT, Symptom Severity subscale of the MDASI-GI-C, and BIPQ were re-administered to evaluate longitudinal changes in psychological distress, symptom burden, and illness perception. Data at T4 were collected through either face-to-face visits or telephone follow-ups. To mitigate potential observation bias, the investigators responsible for data collection were blinded to the primary hypotheses of the study throughout the assessment process.

### 2.4 Statistical analysis

**Figure 1:**
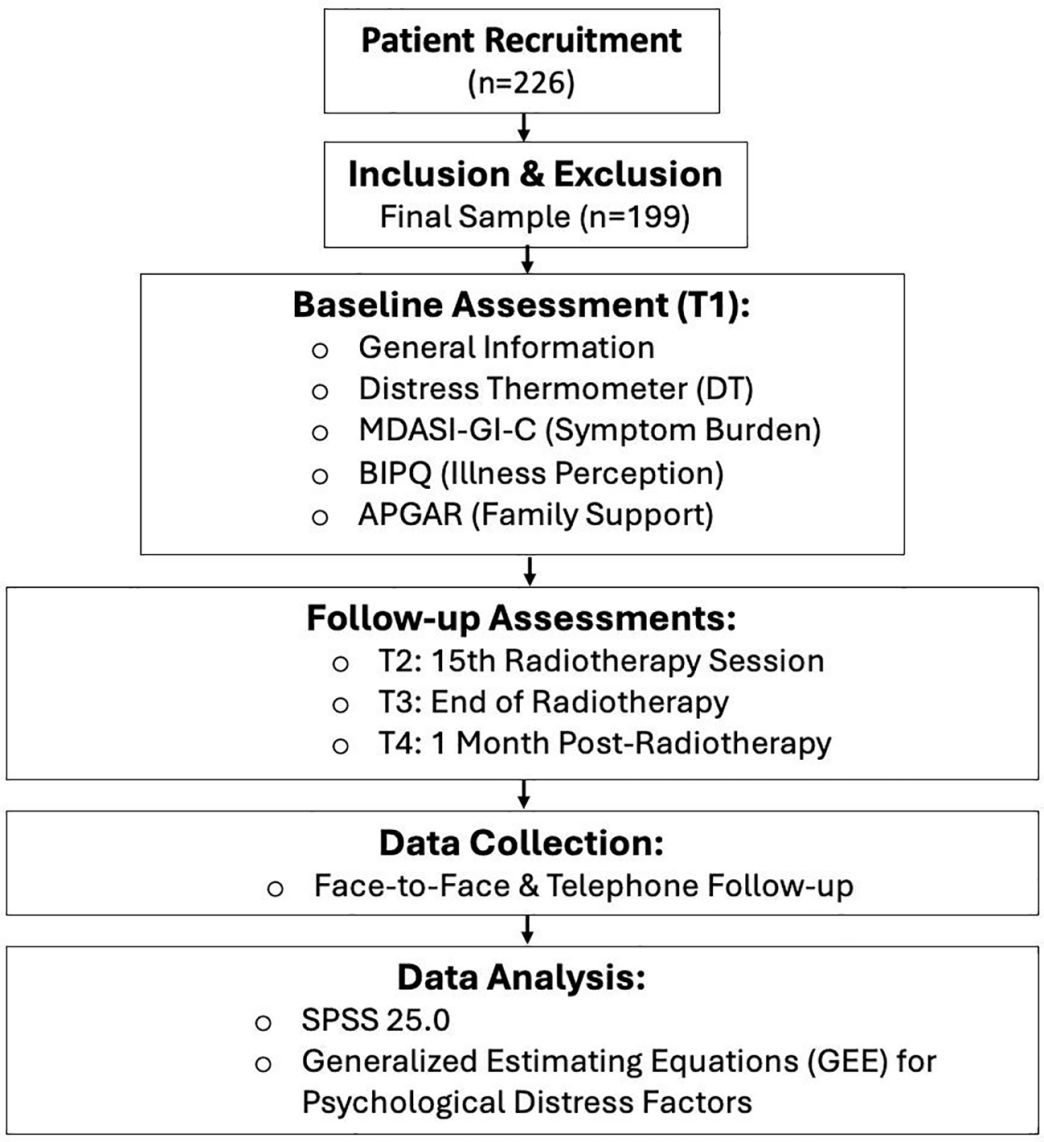
Flowchart of the experimental design.

Data were analyzed using SPSS 25.0. Categorical data are described as frequencies and percentages, with between-group comparisons performed using the chi-square (χ²) test. Continuous data with a normal distribution are expressed as mean ± standard deviation; non-normally distributed data are expressed as median (upper quartile, lower quartile). Repeated measures analysis of variance (ANOVA) was used to compare psychological distress, symptom burden, and illness perception scores across different time points. Multivariate analysis of psychological distress scores was conducted using GEE. The **Figure 1** showed the flowchart of our experimental design.

## 3 Results

### 3.1 Demographic characteristics of esophageal cancer patients undergoing radiotherapy

A total of 226 patients were enrolled and completed the baseline assessment. Of these, 57 (25.22%) were under 60 years of age, 139 (61.50%) were female, 126 (55.75%) had a history of chemotherapy, 84 (37.17%) had undergone prior surgery, and 196 (86.73%) were diagnosed with stage III or IV disease. Further demographic and clinical characteristics are summarized in **Table 1**.

**Table 1:** Demographics of patients treated with radiotherapy for esophageal cancer [n (%)].

Variable	All Participants (n = 226)	Completed follow-up (n = 199)	Lost to follow-up (n = 27)	χ²	P
Age (years)				2.505	0.286
< 60	57 (25.22)	47 (23.62)	10 (37.04)		
60–74	128 (56.64)	116 (58.29)	12 (44.44)		
≥ 75	41 (18.14)	36 (18.09)	5 (18.52)		
Gender				2.310	0.129
Male	139 (61.50)	126 (63.32)	13 (48.15)		
Female	87 (38.50)	73 (36.68)	14 (51.85)		
Monthly Household Income Per Capita (RMB)				0.868	0.648
< 2000	116 (51.33)	104 (52.26)	12 (44.44)		
2000–3000	52 (23.01)	44 (22.11)	8 (29.63)		
> 3000	58 (25.66)	51 (25.63)	7 (25.93)		
Medical Insurance Type				1.845	0.174
Resident Insurance	174 (76.99)	156 (78.39)	18 (66.67)		
Employee Insurance	52 (23.01)	43 (21.61)	9 (33.33)		
Residence				1.203	0.273
Urban	80 (35.40)	73 (36.68)	7 (25.93)		
Rural	146 (64.60)	126 (63.32)	20 (74.07)		
History of Chemotherapy				0.719	0.397
Yes	126 (55.75)	113 (56.78)	13 (48.15)		
No	100 (44.25)	86 (43.22)	14 (51.85)		
History of Surgery				0.168	0.682
Yes	84 (37.17)	73 (36.68)	11 (40.74)		
No	142 (62.83)	126 (63.32)	16 (59.26)		
Tumor Location				4.422	0.219
Cervical	44 (19.47)	41 (20.60)	3 (11.11)		
Upper Thoracic	42 (18.58)	36 (18.09)	6 (22.22)		
Mid-Thoracic	80 (35.40)	73 (36.68)	7 (25.93)		
Lower Thoracic	60 (26.55)	49 (24.62)	11 (40.74)		
Tumor Stage				0.003	0.959
Stage I–II	30 (13.27)	27 (13.57)	3 (11.11)		
Stage III–IV	196 (86.73)	172 (86.43)	24 (88.89)		
Disease Duration				1.243	0.537
< 3 months	108 (47.79)	96 (48.24)	12 (44.44)		
3 months–2 years	84 (37.17)	75 (37.69)	9 (33.33)		
> 2 years	34 (15.04)	28 (14.07)	6 (22.22)		
Concurrent Chemoradiotherapy				0.273	0.601
Yes	136 (60.18)	121 (60.80)	15 (55.56)		
No	90 (39.82)	78 (39.20)	12 (44.44)		

During the follow-up period, 27 patients were excluded from the final analysis due to death (n=1), critical illness (n=1), discontinuation of radiotherapy (n=4), refusal to continue participation (n=13), or loss to follow-up (n=8). Ultimately, 199 patients (88.1%) completed all study phases. Comparative analysis revealed no statistically significant differences in baseline characteristics between patients who completed the study and those who were lost to follow-up (all P > 0.05).

### 3.2 Family APGAR scores

Based on normality testing, the total Family APGAR scores were non-normally distributed. The median score with the interquartile range was 7 (5, 8) (**Table 2**).

**Table 2:** APGAR scores of patients treated with radiotherapy for esophageal cancer [M (P25, P75)].

Instrument	Number of items	Median (IQR)	Score range
APGAR	5	7 (5, 8)	2–10

### 3.3 Psychological distress, symptom burden, and illness perception scores at different time points

The mean psychological distress scores for the 199 patients across the four time points were as follows: T1 (4.88 ± 1.63), T2 (5.09 ± 1.57), T3 (4.75 ± 1.56), and T4 (4.06 ± 1.57). ANOVA revealed a statistically significant difference among the time points (P < 0.05). *Post-hoc* analysis further indicated that psychological distress increased significantly from T1 to T2, followed by a gradual decrease from T2 to T4. Similar trends were also observed for symptom burden and illness perception (**Table 3**).

**Table 3:** Scores at T1–T4 for psychological distress, symptom burden, and illness perception in esophageal cancer patients undergoing radiotherapy.

Measure	T1	T2	T3	T4	F	P	*Post hoc* test
Psychological Distress Score	4.88 ± 1.63	5.09 ± 1.57	4.75 ± 1.56	4.06 ± 1.57	122.928	< 0.001	T2>T1=T3>T4
Symptom Burden	36.36 ± 16.41	50.27 ± 16.46	48.42 ± 14.96	34.60 ± 14.66	202.805	< 0.001	T2>T3>T1=T4
Illness Perception	36.96 ± 6.00	37.91 ± 5.65	34.90 ± 5.68	30.72 ± 5.75	293.183	< 0.001	T2>T1=T3>T4

### 3.4 Factors influencing psychological distress

GEE analysis was performed with psychological distress score as the dependent variable and general characteristics, Family APGAR, symptom burden, and illness perception as independent variables. The results showed that age, monthly household income per capita, residence, tumor stage, concurrent chemoradiotherapy, family support, symptom burden, and illness perception were significant influencing factors (P < 0.05) (**Table 4**).

**Table 4:** Results of GEE multivariate analysis of psychological distress scores in esophageal cancer patients undergoing radiotherapy.

Parameter	B	Sb	95% CI		Wald χ²	P
			LCL	UCL		
Intercept	1.932	0.752	0.459	3.406	6.606	0.010
Age (years)						
< 60	1.104	0.210	0.693	1.516	27.693	<0.001
60–74	0.466	0.175	0.123	0.809	7.109	0.008
≥ 75	—	—	—	—	—	—
Gender						
Male	-0.167	0.132	-0.425	0.091	1.612	0.204
Female	—	—	—	—	—	—
Monthly household income per capita (RMB)						
< 2000	0.826	0.260	0.317	1.335	10.115	0.001
2000–3000	0.386	0.217	-0.039	0.811	3.173	0.075
> 3000	—	—	—	—	—	—
Medical insurance type						
Resident Insurance	0.040	0.147	-0.248	0.328	0.073	0.787
Employee Insurance	—	—	—	—	—	—
Residence						
Urban	0.536	0.192	0.159	0.913	7.775	0.005
Rural	—	—	—	—	—	—
History of chemotherapy						
Yes	-0.121	0.144	-0.403	0.160	0.713	0.398
No	—	—	—	—	—	—
History of surgery						
Yes	0.001	0.155	-0.304	0.303	0.001	0.998
No	—	—	—	—	—	—
Tumor location						
Cervical	0.180	0.187	-0.187	0.548	0.927	0.336
Upper Thoracic	0.019	0.198	-0.369	0.407	0.009	0.923
Mid-Thoracic	-0.062	0.170	-0.395	0.271	0.134	0.714
Lower Thoracic	—	—	—	—	—	—
Tumor stage						
Stage I–II	-0.492	0.136	-0.759	-0.225	13.070	<0.001
Stage III–IV	—	—	—	—	—	—
Disease duration						
< 3 months	-0.334	0.249	-0.822	0.154	1.800	0.180
3 months–2 years	-0.368	0.219	-0.799	0.062	2.819	0.093
> 2 years	—	—	—	—	—	—
Concurrent chemoradiotherapy						
Yes	0.445	0.120	0.211	0.680	13.849	<0.001
No	—	—	—	—	—	—
Symptom Burden	0.034	0.003	0.028	0.040	113.788	<0.001
Family Support	-0.241	0.043	-0.325	-0.156	31.191	<0.001
Illness Perception	0.055	0.009	0.038	0.072	42.082	<0.001

## 4 Discussion

The findings of this longitudinal study reveal a dynamic trajectory of psychological distress among esophageal cancer patients undergoing radiotherapy, characterized by an initial increase followed by a significant decline. Furthermore, our analysis identified a constellation of multifaceted factors significantly influencing psychological distress levels, encompassing sociodemographic characteristics (age, income, residence), clinical factors (tumor stage, concurrent chemoradiotherapy), and psychosocial determinants (family support, symptom burden, illness perception). These findings warrant further discussion.

### 4.1 Overall trajectory of psychological distress

The trajectory of psychological distress in our cohort exhibited an initial increase from T1 to T2, followed by a gradual decline from T2 to T4. A similar pattern of early elevation has been observed in other cancer populations, such as ovarian cancer patients assessed during the peri-chemotherapy period (20). The rise in distress at T2 may be largely attributable to the emergence of radiotherapy-related toxicities, including radiation esophagitis and bone marrow suppression, which compound physical discomfort and functional impairment (2, 3). However, other factors may also contribute to this initial increase. For instance, the early phase of treatment often coincides with heightened uncertainty about treatment efficacy and anxiety regarding potential side effects (9, 13). Additionally, the cumulative financial burden and disruptions to daily routines may further exacerbate psychological distress during this period (11). Notably, the peak symptom burden observed at T2 underscores a critical period where physical and emotional distress converge. This necessitates preemptive management through multimodal support strategies—including structured symptom control, early psychological intervention, and proactive education initiated before T2—to enhance coping capacity and mitigate distress.

### 4.2 Factors influencing psychological distress

#### 4.2.1 Age, monthly income, and residence

Our findings identified younger age as a significant risk factor for psychological distress. Patients under 60 years of age exhibited an average increase of 1.104 points in distress scores compared to those aged 75 years or older, a result consistent with the report by Okereke et al. (21). This association may be attributed to younger individuals’ relatively limited life experience and coping resources, combined with greater familial and social responsibilities. The abrupt interruption of personal and professional trajectories, coupled with potentially lower psychological resilience, may exacerbate distress in this group. In contrast, older patients may possess a more reconciled perspective on health and mortality, contributing to better emotional adaptation (22). These findings highlight the need for targeted interventions—such as resilience-building programs, psychological counseling, and facilitated social support—for younger esophageal cancer patients undergoing radiotherapy.

Our analysis revealed that a lower monthly household income per capita (<2000 RMB) was significantly associated with elevated psychological distress. The substantial financial burden associated with cancer treatment—including direct medical expenses and loss of income—can considerably impair patients’ quality of life (23). In the current therapeutic landscape, where immunotherapy and targeted treatments are increasingly utilized but often involve high costs, economic pressure may further intensify psychological distress. To mitigate this burden in low-income populations, it is essential to enhance accessibility to medical financial assistance, charitable resources, and economic counseling, as well as to improve awareness and utilization of available health insurance benefits.

Interestingly, our analysis indicated that urban residence was associated with higher levels of psychological distress compared to rural residence. This finding aligns with a growing body of evidence suggesting that the urban environment itself can be a source of chronic stress (24). Several sociocultural mechanisms may explain this association. Urban residents often face a higher cost of living and financial pressures, which are well-documented stressors for cancer patients (25). The fast-paced, competitive nature of urban life can lead to social isolation and weaker community bonds, reducing access to buffering social support (26). Furthermore, greater health literacy and awareness among urban populations, while beneficial in some aspects, may also lead to heightened illness-related anxiety and symptom hypervigilance (27). Further investigation is warranted to disentangle the specific sociocultural determinants of distress across diverse residential settings.

#### 4.2.2 Tumor stage, concurrent chemoradiotherapy, and symptom burden

The majority of patients (86.43%) presented with advanced tumor stages (III–IV), a finding consistent with the typical clinical profile of individuals requiring radiotherapy. Patients with advanced disease reported a statistically significant increase in distress scores (average +0.492 points) compared to those in stages I–II. This underscores tumor stage as a critical determinant of symptom burden. While this association is well-established, our data reinforce the urgent need for integrated supportive care early in the management trajectory for this subgroup. The provision of enhanced psychological support—including early palliative care consultation and facilitation of support group participation—should be standardized to improve coping mechanisms and strengthen self-efficacy in disease management (28).

A substantial proportion of the cohort (60.80%) received concurrent chemoradiotherapy (CCRT). As anticipated, these patients exhibited significantly higher levels of psychological distress compared to those undergoing radiotherapy alone. This association, however, requires careful interpretation. Patients selected for CCRT typically present with more advanced disease or poorer prognostic characteristics at baseline, which are themselves known contributors to heightened distress (29). Therefore, the observed effect may reflect a confluence of aggressive treatment and underlying disease severity rather than the sole impact of CCRT. For this high-risk subgroup, the implementation of systematic, pre-emptive symptom management protocols from the initiation of treatment is clinically imperative (30). Future longitudinal studies designed to control for baseline clinical variables are needed to disentangle the specific contribution of the treatment modality itself from tumor-related factors in exacerbating symptom burden and psychological distress.

#### 4.2.3 Family support and illness perception

Our results robustly confirm the pivotal role of psychosocial determinants in the distress experienced by esophageal cancer patients. A higher Family APGAR score, indicative of stronger family functioning, was significantly associated with lower psychological distress. This finding aligns with established theoretical frameworks, such as the Cancer Stress and Coping Model (31), which posit that robust social support systems are critical buffers against illness-related stress. Clinically, this transcends a mere correlation; it implies that family function should be formally assessed as a vital sign of psychosocial vulnerability. For patients from families with low APGAR scores, standard care should be augmented with targeted family-system interventions. This could involve facilitating structured family meetings led by oncology social workers or nurses to improve communication, allocate caregiving responsibilities effectively, and mobilize collective coping resources (32). Viewing the family as a unit of intervention, rather than solely focusing on the patient, represents a critical shift towards more holistic and effective supportive care.

Conversely, a more negative illness perception was a strong predictor of heightened distress. This finding critically underscores that a patient’s subjective cognitive and emotional appraisal of their illness can be as impactful as the physical symptoms themselves. It challenges a purely biomedical approach and argues for the integration of cognitive-behavioral strategies into routine oncology practice (33). For patients with maladaptive illness perceptions, clinicians can implement brief, structured interventions targeting cognitive restructuring. For example, using techniques derived from the Common-Sense Model of self-regulation, healthcare providers can help patients reframe catastrophic thoughts about their prognosis, enhance their understanding of the treatment’s purpose, and bolster self-efficacy in symptom management (34). This proactive approach to addressing illness perceptions is not ancillary but fundamental to improving emotional adjustment and potentially even treatment adherence (35).

### 4.3 Limitations

This study has several limitations that should be considered when interpreting the findings. First, the reliance on a convenience sampling strategy within a single-center setting may restrict the generalizability of the results to broader populations. Second, the use of self-reported measures for assessing psychological constructs carries the risk of common method bias and lacks complementary objective biological indicators. Third, participant attrition over the course of the study, combined with the lack of follow-up psychological data from those who dropped out, limited the ability to conduct longitudinal comparisons between study completers and non-completers. Fourth, potential confounding variables—such as extrafamilial social support and the use of medications (e.g., analgesics or psychotropic drugs)—were not systematically controlled for, which may have influenced the observed outcomes. To address these issues, future research would benefit from employing multi-center randomized sampling designs, integrating objective biomarkers, and incorporating a more comprehensive set of socio-environmental and clinical covariates to strengthen the validity and generalizability of the findings.

## 5 Conclusion

This longitudinal study elucidates the trajectory of psychological distress and its determinants in esophageal cancer patients receiving radiotherapy. The findings offer an empirical basis for implementing targeted supportive care. Specifically, the results highlight the importance of dynamic and personalized interventions, such as stage-tailored health education to improve preparedness and self-management during early treatment phases.

## Data Availability

The original contributions presented in the study are included in the article/supplementary material. Further inquiries can be directed to the corresponding author.
